
JOURNAL INFORMATION
==============================
NLM Title Abbreviation: ScientificWorldJournal
Journal ID: ScientificWorldJournal
NLM Journal ID: 101131163
Journal ID: TSWJ

The Scientific World Journal

ISSN: 2356-6140
EISSN: 1537-744X
Publisher: Wiley

ARTICLE INFORMATION
==============================
PMCID: PMC6084178
PMID: 30147569
DOI: 10.1100/tsw.2001.213
Article ID: 934806
Article version: 1
Subjects: Short Report

CDC25A Mediates Survival by Activating AKT Kinase

Leisser C. 1
Fuhrmann G. 1
Rosenberger G. 1
Grusch M. 1
Halama T. 1
Sasgary S. 2
Cerni C. 2
Krupitza G. 1 *
1 Institute of Clinical Pathology, Waehringer Guertel 18-20, Austria
2 Institute for Cancer Research, Univ. of Vienna, Borschkegasse 8a, A-1090 Vienna, Austria
*G. Krupitza: g.krupitza@akh-wien.ac.at
Publication date: 2001
Electronic publication date: 2001 Oct 12
Volume: 1
First page: 94
Last page: 94
Copyright: Copyright © 2001 C. Leisser et al.
Copyright year: 2001
License: This is an open access article distributed under the Creative Commons Attribution License, which permits unrestricted use, distribution, and reproduction in any medium, provided the original work is properly cited.
License URL: https://creativecommons.org/licenses/by/3.0/

Funding: Austrian National Bank Fund 7220 Herzfelder’sche Familienstiftung Anton Dreher Memory Foundation

==============================
Miami *Nature Biotechnology* Short Reports
*TheScientificWorld* (2001) 1 (S3), 94SR
ISSN 1532-2246; DOI 10.1100/tsw.2001.213

### CDC25A MEDIATES SURVIVAL BY ACTIVATING AKT KINASE

C. Leisser[1], G. Fuhrmann[1], G. Rosenberger[1], M. Grusch[1], T. Halama[1], S. Sasgary[2], C. Cerni[2], G. Krupitza[1]*

[1]Institute of Clinical Pathology, Waehringer Guertel 18-20; [2]Institute for Cancer Research. Univ. of Vienna, Borschkegasse 8a, A-1090 Vienna, Austria.
* g.krupitza@akh-wien.ac.at

**INTRODUCTION.** The cell cycle specific phosphatase Cdc25A was shown to be down regulated before onset of Myc-dependent and chemically induced apoptosis (1, 2). Also during apoptosis elicited by serum withdrawal, Cdc25A-repression is observed and FGF- and PDGF-dependent survival re-establishes Cdc25A levels. We demonstrate that Cdc25A mediates FGF-survival signals to Akt.

**METHODS.** Northern-, Western-blotting, DNA-analysis, TUNEL assay (1,2) and HO/PI double staining (3), transfection and retroviral transduction were performed according to standard protocols. Immunoprecipitation in conjunction with Western blot analysis: Cells were lysed in buffer containing 20-mM HEPES pH 7.9, 0.4 M NaCl, 2.5% glycerol, 1 mM EDTA and protease inhibitors by freeze-thaw cycle(s) in liquid nitrogen and antibody antigen complexes were precipitated by protein G sepharose.

**RESULTS.** Ectopic overexpression of Cdc25A renders the rat embryonic cell line 423 refractory to apoptosis that is induced by serum deprivation. This effect is specific because it can be reverted by a conditional Cdc25A-antisense construct.

In Cdc25A overexpressing 423 cells increased levels of serine 473-phosphorylated Akt are found (Fig. 1). To identify the signaling proteins mediating the survival function to Cdc25A, Cdc25A- and akt- overexpressing pooled clones were exposed to selected chemicals (SU4984, NF023, Rapamycin) that inhibit defined signaling pathways (Tab. 1). The obtained data locate Cdc25A- and Akt downstream of FGF-receptor function G-protein-, and PP2A- activity (respectively) and suggest that Akt and Cdc25A are within the same pathway. In fact, Akt and Raf1, which are two prominent components for survival signaling, co-precipitate together with Cdc25A as a trimeric complex (Fig. 2).

**Fig. 1**

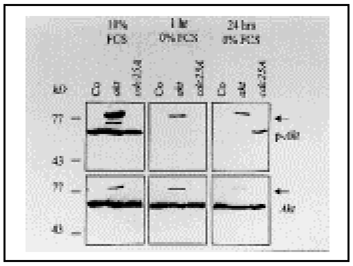

**Fig. 2**

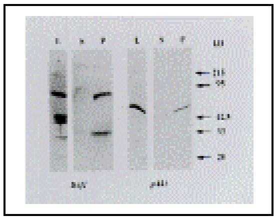

**Tab. 1**
Survival rate of controls, Cdc25A- and Akt- over-expressing clones modulated by inhibitors of signal transduction.

| inhibitor | mock | *cdc25A* | *akt* |
|---|---|---|---|
| | | | |
| SU4984 | | | |
| 5 uM | 88,2 | 98,8 | 101,2 |
| 10 uM | 83,8 | 93,9 | 101,0 |
| 20 uM | 78,4 | 94,4 | 94,1 |
| NF023 | | | |
| 0,5 uM | 91,6 | 102,8 | 97,1 |
| 1,0 uM | 88,5 | 101,8 | 98,3 |
| 2,0 uM | 84,2 | 102,1 | 102,5 |
| LY294002 | | | |
| 5 uM | 67,9 | 70,6 | 71,5 |
| 10 uM | 58,3 | 59,9 | 61,8 |
| 20 uM | 53,7 | 55,9 | 56,1 |
| Rapamycin | | | |
| 1 nM | 87,8 | 100,3 | 98,7 |
| 5 nM | 82,1 | 95,1 | 96,5 |
| 10 nM | 80,1 | 94,8 | 93,7 |

**CONCLUSION.** Whereas it was shown that apoptosis of Val5 and 3T3LI cells is dependent on Cdc25A expression (4), N.1 ovarian carcinoma cells suppress Cdc25A protein level prior apoptosis (1, 2). Ectopic expression of Cdc25A in rat 423 cells prevents apoptosis due to the phosphorylation of Akt. Akt-activation is presumably achieved by Raf1, which co-precipitates in the same multi-protein complex with Cdc25A and Akt. It was shown that Raf1 becomes de-phosphorylated and activated by Cdc25A. We assume that the pro-apoptotic activity of Cdc25A observed by others (4) in Val5 and 3T3LI cells might be exerted due to nuclear and cell cycle-specific function. In contrast, the majority of Cdc25A in rat 423 cells is located in the cytoplasma, which also explains interaction with Raf1 and Akt, two proteins acting close to the cellular membrane.

**ACKNOWLEDGEMENT.** This work was supported by the Austrian National Bank Fund No.: 7220, the Herzfelder´sche Family Foundation, and the Anton Dreher Memory Foundation.

**REFERENCES.**

1. Krupitza, G., Grusch, M., Braun, K., Fuhrmann, G., Steinbrugger, R., Hulla, W., Simonitsch, I., Chott, A., and Hengstschläger, M. (1998) Cell Death Differ. 5, 758-764
2. Grusch, M., Rosenberger, G., Fuhrmann, G., Braun, K., Titscher, B., Szekeres, T., Fitzer-Szekeres, M., Oberhuber, G., Krohn, K., Hengstschläger, M., Krupitza, G., and Jayaram, H. (1999) Cell Death Differ. 6, 736-744
3. Fritzer-Szekeres, M., Grusch, M., Luxbacher, C., Horvath, S., Krupitza, G., Elford, H.L., and Szekeres, T. (2000) Exp. Hematol. 28, 924-930
4. Galaktionov, K., Chen, X., and Beach, D. (1996) Nature 382, 511-517
